# Are we entering a new era for qualitative research? Using qualitative evidence to support guidance and guideline development by the World Health Organization

**DOI:** 10.1186/s12939-018-0841-x

**Published:** 2018-09-24

**Authors:** Simon Lewin, Claire Glenton

**Affiliations:** 1 Norwegian Institute of Public Health, PO Box 222 Skøyen, 0213 Oslo, Norway; 2 Health Systems Research Unit, South African Medical Research Council, Cape Town, South Africa; 3 Cochrane EPOC Group, Norwegian Institute of Public Health, Oslo, Norway; 4 Cochrane Norway, Norwegian Institute of Public Health, PO Box 222 Skøyen, 0213 Oslo, Norway

**Keywords:** Qualitative research, Qualitative evidence synthesis, Social science approach, Evidence ecosystem, Guidelines, Health systems, Systematic review, Capacity strengthening, Decision making, Policy making

## Abstract

Qualitative approaches are one of several methodologies utilised within the social sciences. New developments within qualitative methods are widening the opportunities for using qualitative evidence to inform health policy and systems decisions. In this commentary, we discuss how, in our work with the World Health Organization (WHO), we have explored ways of broadening the types of evidence used to develop evidence-informed guidance for health systems.

Health systems decisions are commonly informed by evidence on the effectiveness of health system interventions. However, decision makers and other stakeholders also typically have additional questions, including how different stakeholders value different outcomes, the acceptability and feasibility of different interventions and the impacts of these interventions on equity and human rights. Evidence from qualitative research can help address these questions, and a number of WHO guidelines are now using qualitative evidence in this way. This growing use of qualitative evidence to inform decision making has been facilitated by recent methodological developments, including robust methods for qualitative evidence syntheses and approaches for assessing how much confidence to place in findings from such syntheses. For research evidence to contribute optimally to improving and sustaining the performance of health systems, it needs to be transferred easily between different elements of what has been termed the ‘evidence ecosystem’. This ecosystem includes primary and secondary evidence producers, guidance developers and those implementing and evaluating interventions to strengthen health systems. We argue that most of the elements of an ecosystem for qualitative evidence are now in place – an important milestone that suggests that we are entering a new era for qualitative research. However, a number of challenges and constraints remain. These include how to build stronger links between the communities involved in the different parts of the qualitative evidence ecosystem and the need to strengthen capacity, particularly in low and middle income countries, to produce and utilise qualitative evidence and decision products informed by such evidence. We invite others who want to support the wider use of qualitative evidence in decision processes to look for opportunities in their settings to put this into practice.

The growing use of qualitative evidence to support decisions, and the availability of methods that can help us use this type of evidence in knowledge-to-action cycles [1, 2], suggest that we are entering a new era for qualitative research. Qualitative approaches are one of many methodologies utilised within the social sciences. Here we focus here on how new developments in the field of qualitative research are creating important opportunities for using qualitative evidence, including findings from syntheses of qualitative evidence, to inform health policy and systems decisions.

Health systems are complex, creating challenges for decision makers aiming to strengthen these systems and achieve the Sustainable Development Goals [3]. Most stakeholders agree, however, that decisions about which interventions or policy options to implement should be informed by the best available global and local evidence [4, 5]. Within clinical care there is a long history of using evidence-based guidelines to inform decisions. However, evidence-based health systems guidance is a more recent development. Health systems guidance has been defined as “systematically developed statements produced at global or national levels to assist decisions about appropriate options for addressing a health systems challenge in a range of settings and to assist with the implementation of these options and their monitoring and evaluation” [6]. Health systems guidance can address issues such as options for funding national lay health worker programmes; which digital interventions might effectively support health care delivery; and ways of retaining health care providers in rural areas.

In our work with the World Health Organization (WHO) we have explored ways of broadening the types of evidence that are used to develop health systems guidance [7, 8]. While questions of effectiveness are central to health systems decisions, decision makers also want to know more about how different stakeholders value different outcomes, the acceptability and feasibility of different interventions and the impacts of these interventions on equity and human rights [6, 7, 9]. Evidence from qualitative research can play a key role in addressing these considerations [8]. This is because well-designed qualitative research allows us to explore how people experience and conceptualise the world around them, including health systems and services; and can help us understand how and why these systems succeed or fail. For instance, a recent qualitative study of introducing and implementing humanised childbirth care in a referral hospital in Benin highlighted some of the challenges experienced by midwives and other health care providers in delivering such care, and developed a conceptual model of humanised care in this setting [10]. This primary study was, in turn, incorporated into a qualitative evidence synthesis (or systematic review of qualitative studies) of factors that influence the provision of childbirth care by skilled birth attendants in low and middle income countries [11]. This synthesis subsequently informed a WHO statement on skilled health personnel providing care during childbirth [12]. In addition to the WHO, a range of other organisations involved in producing guidance and health technology assessments are also increasingly using qualitative evidence to answer questions related to, for instance, the acceptability and feasibility of interventions. These organisations include the National Institute of Health and Clinical Excellence (NICE) in the United Kingdom [13, 14], the Swedish Public Health Institute and the South African Fetal Alcohol Spectrum Disorders Task Team [15].

One example of the use of qualitative evidence to inform WHO guidance is the recent set of WHO recommendations on ‘Antenatal care for a positive pregnancy experience’ [16]. These include recommendations on health systems interventions to improve the quality of antenatal care and women’s use of this care. To ensure that women’s perspectives shaped the development of this guidance, the WHO first commissioned a qualitative evidence synthesis that gathered studies from across the world exploring what women want, need and value in pregnancy [17]. The WHO used this synthesis when determining the broader aims of the guidance and the key outcomes to be considered when gathering evidence and making recommendations. For instance, the concept of a “positive pregnancy experience” became the core focus of the guidance as a means of ensuring that person-centred health and well-being was prioritized. Also, the outcome “positive pregnancy experience” was included for most guidance questions, ensuring that each intervention was evaluated against this key issue for women. Following the scoping stage of the guidance, the WHO commissioned a second qualitative evidence synthesis to explore factors influencing women’s use of antenatal services [18]. These findings fed into the guidance process by answering questions about the acceptability and feasibility of the interventions to women and other stakeholders. A number of other recent WHO guidelines have used similar approaches [19–21].

The growing use of qualitative evidence to inform decision making has been facilitated by a number of key developments in the field, including better standards for reporting primary qualitative studies [22, 23]; robust methods for undertaking qualitative evidence syntheses [24]; databases for rapidly identifying such syntheses within the health field [25, 26]; the emergence of GRADE-CERQual - an approach for assessing how much confidence to place in findings from qualitative evidence syntheses [27, 28]; and frameworks that facilitate the packaging of different types of evidence to facilitate transparent and systematic assessment by decision makers [29, 30]. The challenge now is to mainstream these efforts so that qualitative evidence, ideally from syntheses of primary qualitative studies, is used more widely to develop health systems guidance and clinical guidelines within WHO and within other guideline development organisations. In our experience, one important constraint includes identifying teams to conduct policy-relevant qualitative evidence syntheses, particularly in low and middle income countries. We also need to explore further how to help members of guideline panels and other decision makers engage with different types of evidence and make judgements about these when formulating recommendations. In addition, we need to expand efforts to strengthen capacity in low and middle income countries to undertake health policy and systems relevant primary qualitative research.

For research evidence to contribute optimally to improving and sustaining the performance of health systems, it needs to be transferred easily between different elements of what has been termed the ‘evidence ecosystem’ [31–33]. This ecosystem includes those producing primary evidence and those synthesising the evidence; people producing evidence-informed decision products such as health systems guidance and clinical practice guidelines; those responsible for implementing evidence-informed options within health systems, including programme managers and decision makers; and those involved in delivering and using health services, including service providers, service users and citizens [31]. Recent developments within the field of qualitative research, including those described above, mean that we now have most of the elements of an ecosystem for qualitative evidence in place. Evidence from primary qualitative studies is now being gathered in evidence syntheses; the findings of these syntheses are being used in decision products such as guidance and policy briefs [34, 35]; and decision products informed by qualitative evidence are being used to guide choices on health system options and, in turn, are informing choices by service providers and users. Finally, these health system strengthening initiatives are being evaluated through new primary qualitative research [36, 37]. In addition, we now have a better understanding of how primary qualitative research should be designed to meet the needs of those synthesising research and decision makers.

Having the elements of a qualitative evidence ecosystem in place is an important milestone and suggests that we are entering a new and exciting era within the field of qualitative research. Of course, a number of challenges and constraints remain, as we have noted earlier. Other challenges include how to build stronger links between the communities involved in the different parts of the qualitative evidence ecosystem, including across all sectors relevant to the Sustainable Development Goals [3], and the need to strengthen capacity across settings and institutions, particularly in low and middle income countries, to produce and utilise qualitative evidence and decision products informed by such evidence. Our experience of working with the WHO has helped us to collaboratively develop methods for using qualitative evidence in guideline and guidance development. We realise, however, that we are also not yet making full use of the potential of qualitative evidence synthesis findings to shape the development of implementation considerations for guidance or to inform guidance contextualisation, adaptation and implementation processes at national and sub-national levels. This is in part because we need to explore both ways of integrating these findings with local evidence [4], including the knowledge and experience of local stakeholders, and approaches for working with local stakeholders to develop implementation options. Efforts are also needed to strengthen the capacity of local stakeholders to understand and use qualitative evidence. These are areas in which we and others are now doing methodological research.

As we take this work forward, we should also not forget what is perhaps the most important role that qualitative evidence can play in decision-making: representing the views and experiences of stakeholders, including vulnerable and marginalised groups who are often not represented directly. By drawing on the global body of qualitative evidence, qualitative evidence syntheses have the potential, when used together with direct stakeholder engagement, to help ensure that decisions are guided by stakeholders’ views and that these decisions do not widen inequities. As we have noted elsewhere, using qualitative evidence in this way may also contribute to increased transparency and accountability in public decision-making [27].

Our experiences of working with the WHO have taught us that the best way of learning is doing. We support Health Systems Global’s efforts to promote both primary and secondary social science research on health policies and systems, and to strengthen capacity and collaborations in this area [38]. However, we need to go further than producing more social science research that is policy relevant and is conducted in ways that address the tensions and complexities involved in commissioning and undertaking research across different settings and groups [39, 40]. As a social science research community, we also need to work more closely with policy users and other stakeholders to build capacity for evidence use. We therefore invite others who believe that we need greater recognition of the value of qualitative research, and who want to support the wider use of qualitative evidence in decision processes, to look for opportunities in their settings to put these beliefs into practice.
